# The Hydroalcoholic Extract Obtained from *Mentha piperita* L. Leaves Attenuates Oxidative Stress and Improves Survival in Lipopolysaccharide-Treated Macrophages

**DOI:** 10.1155/2017/2078794

**Published:** 2017-09-20

**Authors:** Mariana Oliveira Arruda, Saulo José Figueiredo Mendes, Simone Aparecida Teixeira, Ludmilla Santos Silva de Mesquita, Maria Nilce de Sousa Ribeiro, Stanley de Sousa Lima Galvão, Marcelo Nicolás Muscará, Elizabeth Soares Fernandes, Valério Monteiro-Neto

**Affiliations:** ^1^Programa de Pós-graduação, Universidade CEUMA, Rua Josué Montello, No. 1, Renascença, 65075-120 São Luís, MA, Brazil; ^2^Departamento de Farmacologia, Universidade de São Paulo, Av. Prof. Lineu Prestes, No. 1524 Sala 326, Butantan, 05508-900 São Paulo, SP, Brazil; ^3^Laboratório de Farmacognosia, 65080-805 São Luís, MA, Brazil; ^4^Departamento de Patologia, Universidade Federal do Maranhão, Avenida dos Portugueses, No. 1966, Bacanga, 65080-805 São Luís, MA, Brazil

## Abstract

*Mentha piperita* L. (peppermint) possesses antimicrobial properties, but little is known of its ability to modulate macrophages. Macrophages are essential in bacterial infection control due to their antimicrobial functions and ability to link the innate and adaptive immune responses. We evaluated the effects of the peppermint leaf hydroalcoholic extract (LHAE) on cultured murine peritoneal macrophages stimulated or not with lipopolysaccharide (LPS) *in vitro*. Vehicle-treated cells were used as controls. The constituents of the extract were also identified. Epicatechin was the major compound detected in the LHAE. LPS-induced macrophage death was reversed by incubation with LHAE (1–30 *μ*g/ml). Higher concentrations of the extract (≥100 *μ*g/ml) decreased macrophage viability (49–57%) in the absence of LPS. LHAE (1–300 *μ*g/ml) attenuated H_2_O_2_ (34.6–53.4%) but not nitric oxide production by these cells. At similar concentrations, the extract increased the activity of superoxide dismutase (15.3–63.5-fold) and glutathione peroxidase (34.4–73.6-fold) in LPS-treated macrophages. Only LPS-unstimulated macrophages presented enhanced phagocytosis (3.6–6.6-fold increase) when incubated with LHAE (3–30 *μ*g/ml). Overall, the LHAE obtained from peppermint modulates macrophage-mediated inflammatory responses, by stimulating the antioxidant pathway in these cells. These effects may be beneficial when the excessive activation of macrophages contributes to tissue damage during infectious disease.

## 1. Introduction

Macrophages are on the first line of the host's immune response to bacterial infection. Indeed, these cells play detrimental roles in pathogen recognition, bacterial killing, and antigen presentation, leading to further activation of adaptive immune responses (see for review [1–3]). Gram-negative bacterial strains are major pathogens causatives of severe infectious diseases in humans, associated with high mortality rates [4, 5]. This is due not only to their ability to become resistant to the available antimicrobials [4] but also depends on an effective macrophage response to these pathogens [6].

The production of oxidant species by macrophages is a hallmark of the inflammatory response to infection (see for review [7, 8]). Oxidant species such as hydrogen peroxide (H_2_O_2_) and superoxide (O_2_^−^) are produced following phagocytosis of the pathogen by these cells as part of their machinery to respond to harmful insults [9]. Alongside an excessive nitric oxide (NO) production, increased levels of prooxidant species may lead to damage and poor perfusion of vital organs of the host, contributing to multiple organ failure; thus, to counteract this response, antioxidant pathways are activated [10].

Natural antioxidants including phenolic compounds have been identified in a variety of plants. Additionally, antimicrobial properties have been attributed to these compounds, suggesting them to be potential therapies for bacterial infections. *Mentha piperita* L., a member of the family Lamiaceae and popularly known as peppermint, is native to the Mediterranean region and has been spread worldwide due to its medicinal properties, taste, and aroma [11]. Its medicinal properties include antitumor, antimicrobial, and antioxidant actions and have been reported especially for its essential oil [12–17]. Of importance, *M. piperita* essential oil was previously shown to be effective against Gram-negative and Gram-positive bacteria and to act as a potential antioxidant *in vitro* [12]. This essential oil was also shown to reduce the numbers of leukocytes in a murine model of skin inflammation [18] and modulate cytokine production *in vivo* [19]. However, the underlying mechanisms of the effects of *M. piperita* on macrophages remain unclear. Considering peppermint antioxidant and anti-inflammatory potentials, we hypothesized whether its leaf hydroalcoholic extract (LHAE) is able to modulate macrophage-mediated inflammatory responses. Therefore, the aim of this study was to investigate the effects of the peppermint leaf hydroalcoholic extract (LHAE) on cultured murine peritoneal macrophages *in vitro*.

## 2. Material and Methods

### 2.1. Plant

The leaves of *M. piperita* were collected in September at Santa Luzia, Maranhão, Brazil (4°4′8″S, 45°41′24″W). A voucher specimen (number 01275) was deposited in the herbarium Ático Seabra of the Federal University of Maranhão, São Luís, Brazil.

### 2.2. Preparation of the Crude Hydroalcoholic Extract

The collected leaves were washed in running water before being dried under forced air circulation at 45°C. The dried leaves were triturated, and the resulting powder was macerated for 10 days in 70% ethyl alcohol (Sigma-Aldrich, St. Louis, MO, USA) at room temperature. The mixture was filtered through cellulose filter paper (Whatman No. 4, GE Healthcare UK, Amersham, UK) and evaporated to dryness under reduced pressure using a rotary evaporator (Eyela N-1200BV-W, Tokyo, Japan) at 40°C. The residual solvent was removed in a vacuum centrifuge at 40°C to yield crude ethanol extracts of leaves.

### 2.3. Chemical Characterization by High-Performance Liquid Chromatography (HPLC)

For HPLC analysis, the peppermint LHAE was dissolved in methanol and water to a final concentration of approximately 5 mg/ml and filtered through a 0.22 *μ*m nylon filter. An HPLC (Surveyor Plus/Finnigan) coupled to an ultraviolet-visible detector (HPLC-UV-Vis), with an ACE 5 C18 reverse phase analytical column (250 × 4.60 mm, 5 *μ*m, ACE) protected by a C18 precolumn (4 × 3 mm, 5 *μ*m, Gemini, Phenomenex), was used for the analysis. Compounds were separated at room temperature using an elution gradient at a flow rate of 0.6 ml/min. Mobile phases consisted of purified water containing 0.1% acetic acid (A) and methanol (B). The following gradient was used: 0–2 min, 5% B; 2–10 min, 25–40% B; 10–20 min, 40–50% B, 20–30 min, 50–60% B; 30–40 min, 60–70% B; and 40–50 min, 70–80% B. Injection volume was 10 *μ*l and UV-Vis detection was performed at 254 nm. Ursolic acid, epicatechin, caffeic acid, rutin, quercetin, naringenin, and kaempferol standards were diluted and analyzed under the same conditions.

### 2.4. Macrophage Assays

#### 2.4.1. Animals

Nonfasted outbred male Swiss mice (2-3 months old) were used. Mice were obtained from the animal's facility of the Universidade CEUMA (UNICEUMA). Mice were kept in a climatically controlled environment (room temperature of 22 ± 2°C and humidity of around 60%) under 12 : 12 h light-dark cycle (lights on 07:00 h). All procedures were approved by the Ethics Committee of UNICEUMA and carried out in accordance with the Brazilian Society for Animal Welfare (SBCAL).

#### 2.4.2. Macrophage Culture and Viability

Peritoneal cells were collected from animals injected intraperitoneally with 1 ml of phosphate-buffered saline (PBS, Sigma-Aldrich, Brazil) containing 1% oyster glycogen (Sigma-Aldrich, Brazil). Briefly, 18 h following injection, the peritoneal cavity was washed with 10 ml of cold PBS and the peritoneal cells were harvested, centrifuged (10 min, 4°C), and resuspended (final concentration of 2 × 10^6^ cells/ml) in DMEM-Glutamax® (Life Technologies, Brazil) containing 10% FCS (v/v, Life Technologies, Brazil) and 1x penicillin-streptomycin (Sigma-Aldrich, Brazil). Cells (6 × 10^5^/well) were incubated in 96-well plates, at 37°C under 5% CO_2_, and after 2 h, nonadhered cells were removed and the adherent cells (macrophages) were incubated with either peppermint LHAE (1–300 *μ*g/ml) or vehicle (1% dimethyl sulfoxide (DMSO, Sigma-Aldrich, Brazil) in PBS), and after 15 min, stimulated with *K. pneumoniae* lipopolysaccharide (LPS, 100 ng/ml in PBS, Sigma-Aldrich, Brazil) or PBS for 24 h. After this period, the supernatant was collected and stored at −80°C for further analysis of NO_2_^−^/NO_3_^−^ (NO end products) and H_2_O_2_ concentrations. For analysis of macrophage viability, the remaining cells were incubated with PrestoBlue® reagent (1 : 10, Life Technologies, Brazil) for 90 min; and then, the absorbances were read at 550 and 650 nm. Results were calculated according with the manufacturer's instructions and are expressed as absorbance in percentage (%) of cell viability in relation to vehicle/PBS-treated cells.

#### 2.4.3. Phagocytosis

In a separate series of experiments, peritoneal macrophages were obtained and cultured (6 × 10^5^/well) as described above in eight chamber culture slides (BD Falcon). Just after removal of nonadherent cells, macrophages were incubated with 2 *μ*m fluorescent latex beads (1 : 100; 5 *μ*l/well; Sigma-Aldrich, Brazil), for 24 h as described by Fernandes et al. [20]. After the incubation period, the cell culture medium was removed and each well was washed three times with PBS. Wells were fixed in 2% paraformaldehyde for 10 min and washed three times with PBS for the removal of excessive paraformaldehyde. Then, 10 *μ*l PBS were added per well and slides were covered with a glass slip. Slides were analyzed in a fluorescence microscope (Zeiss Axio Image Z2, German, ×40 objective, bright field). Two lots of 100 cells were counted for each well, and the average for each well was considered as an *n* number. Results are expressed as percentage of cells containing beads and number of phagocytosed beads per 100 cells.

#### 2.4.4. NO End Product (Nitrate NO_3_^−^ plus Nitrite NO_2_^−^) Measurement

The NO_2_^−^/NO_3_^−^ content was measured by the Griess reaction assay as an indicator of NO production in supernatant samples as previously described [21]. NO_3_^−^ was reduced to nitrite (NO_2_) by incubating 80 *μ*l of the sample with 20 *μ*l of 1 U/ml nitrate reductase and 10 *μ*l of 1 mM NADPH for 30 min at 37°C in a 96-well plate. Next, 100 *μ*l Griess reagent (5% v/v H_3_PO_4_ containing 1% sulfanilic acid and 0.1% N-1-napthylethylenediamine) was added and incubated for 15 min at 37°C. Absorbance at 550 nm was immediately measured using a spectrophotometer (Plate reader MB-580; Heales, Shenzhen, China). After subtraction of background readings, the absorbance in each sample was compared with that obtained from a sodium nitrite (0–100 *μ*M) standard curve and expressed as NO^x^ concentrations (*μ*M).

#### 2.4.5. Measurement of H_2_O_2_ Concentrations

H_2_O_2_ production by macrophages was measured by using a H_2_O_2_/peroxidase assay kit (Amplex Red H_2_O_2_/Peroxidase assay kit, Invitrogen, Brazil), as described by Mendes et al. [21]. Briefly, 50 *μ*l of the supernatants were incubated with 50 *μ*l of a 0.05 M NaPO_4_ (pH 7.4) solution containing 0.2 U/ml horseradish peroxidase (HRP) and 25.7 mg/ml Amplex Red reagent (10-acetyl-3,7-dihydroxyphenoxazine) for 2 h, at 37°C. Samples incubated with 0.05 M NaPO_4_ only were used as controls. After incubation, the absorbance was read at 560 nm. After subtraction of background readings, the absorbance in each sample was compared with that obtained from a H_2_O_2_ standard curve (0–40 *μ*M). H_2_O_2_ concentrations are expressed in *μ*M.

#### 2.4.6. Antioxidant Enzyme Activities

*(1) Sample Preparation*. In another series of experiments, macrophages were obtained, isolated, cultured (6 × 10^5^/well) in 24-well plates, and stimulated as described above. Following incubation with LPS (24 h), the supernatant was removed and 500 *μ*l of 0.05 M NaPO_4_ (pH 7.4) (containing ethylenediaminetetraacetic acid (EDTA), 1 mM) was added to each well. Plates were placed on ice for 15 min. Then, cells were scraped from each well, transferred to tubes, and lysed by three snap freezing/defrosting times. Tubes were centrifuged at 10,000 ×g for 10 min at 4°C, and the supernatants were used for the enzyme activity assays.

*(2) Superoxide Dismutase (SOD) Activity Assay*. SOD activity was measured as described by Ukeda et al. [22], with modifications. Briefly, 20 *μ*l of sample were incubated with 200 *μ*l of a solution containing 2.5 ml sodium carbonate buffer (50 mM; pH 9.4) and 0.1 ml of a mixture containing xanthine (3 mM), EDTA (3 mM) and 2,3-Bis-(2-Methoxy-4-Nitro-5-Sulfophenyl)-2H-tetrazolium-5-carboxanilide (XTT, 153 mU/ml), in the presence and absence of SOD. Samples (200 *μ*l/well) were added in 96-well plates and the absorbance was read at 470 nm for 20 min. Results are expressed as milliunits (mU) of SOD/mg of protein. One unit of SOD was defined as the amount of enzyme capable of dismutating 1 *μ*mol of O_2_^−^/min.

*(3) Glutathione Peroxidase (GPx) Activity Assay*. GPx activity was determined as described by Paglia and Valentine [23]. For this, 30 *μ*l of sample per well (diluted 1 : 3) was incubated for 5 min at 37°C, with 145 *μ*l per well of 0.05 M phosphate buffer (pH 7.4) containing 0.1 M EDTA, 5 *μ*l of glutathione (GSH, 80 mM), and 5 *μ*l glutathione reductase (0.0096 U/*μ*l). After incubation, 5 *μ*l of 0.46% *tert*-butyl hydroperoxide solution and 10 *μ*l of 1.2 mM NADPH were added to each well. Absorbances were monitored at 340 nm for 10 min. The results are expressed as *μ*mol of GSH/min/mg of protein.

### 2.5. Statistical Analysis

Data are expressed as mean ± standard error (SEM). Differences between groups were analysed by two-way analysis of variance (ANOVA), followed by Bonferroni's multiple comparison tests, or paired *t*-test as appropriate. Percentages of inhibition were calculated as the mean of the inhibitions obtained for each individual experiment. *p* values < 0.05 were considered statistically significant.

## 3. Results

### 3.1. Chemical Analysis

**Figure 1:**
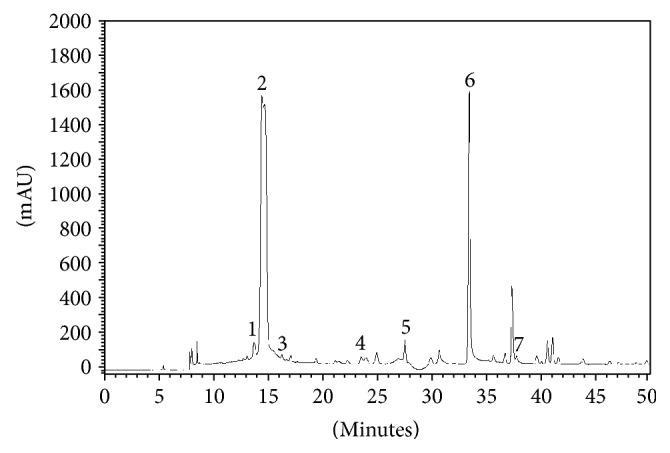
HPLC analysis of the peppermint LHAE. Peaks are numbered 1–7 and were shown to coelute with (1) ursolic acid, (2) epicatechin, (3) caffeic acid, (4) rutin, (5) quercetin, (6) naringenin, and (7) kaempferol.

HPLC analysis of peppermint LHAE detected the presence of seven peaks that coeluted with ursolic acid, epicatechin, caffeic acid, rutin, quercetin, naringenin, and kaempferol (Figure 1). Epicatechin and naringenin were the major compounds, with retention times of 14.5 min and 33.5 min, respectively (Table 1).

### 3.2. Peppermint LHAE Modulates Macrophage Viability

**Figure 2:**
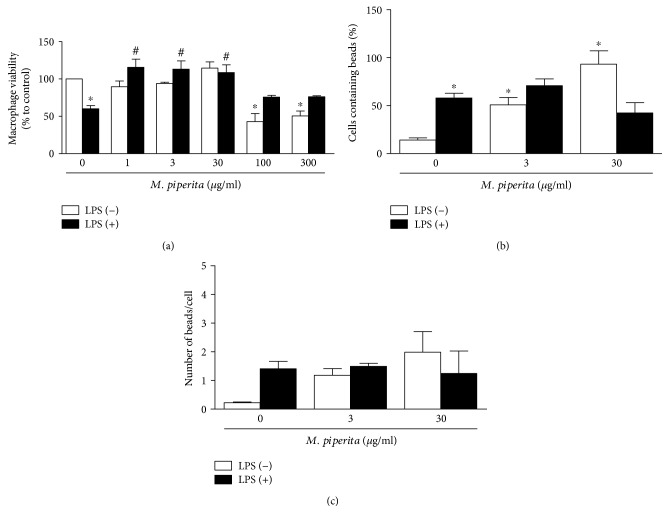
Effect of peppermint LHAE on peritoneal macrophage viability and phagocytosis. Cell viability (a), number of cells containing beads (b), and number of beads per cell (c) were quantified on peritoneal macrophages pretreated with peppermint LHAE (1–300 *μ*g/ml, in 1% DMSO in PBS) and stimulated with LPS for 24 h. Vehicle-treated cells were used as controls. Data are expressed as mean ± SEM. ^∗^*p* < 0.05 compared with vehicle-treated cells; ^#^*p* < 0.05 compared with LPS-treated cells.

Peppermint LHAE effects were evaluated on macrophage viability stimulated or not with LPS. As expected, LPS reduced macrophage viability by 40% (Figure 2(a)). LPS-induced macrophage death was reversed by incubation with LHAE (1–30 *μ*g/ml; Figure 2(a)). At higher concentrations (≥100 *μ*g/ml), the extract decreased (49–57%) the viability of macrophages cultured in the absence of LPS (Figure 2(a)).

### 3.3. Macrophage-Mediated Phagocytosis

LPS stimulated phagocytosis in comparison with vehicle-treated cells, as denoted by an increase in the percentage of cells containing beads (4.1-fold increase) and in the number of beads per cell (6.2-fold increase; Figures 2(b) and 2(c)). Peppermint LHAE potentiated the ability of macrophages to phagocytose in the absence but not in the presence of LPS (Figures 2(b) and 2(c)). This potentiation was as high as 6.6- and 8.8-fold for the percentage of macrophages containing beads and number of beads per cell, respectively (Figures 2(b) and 2(c)).

### 3.4. Peppermint LHAE Reduces H_2_O_2_ but Not NO Production

**Figure 3:**
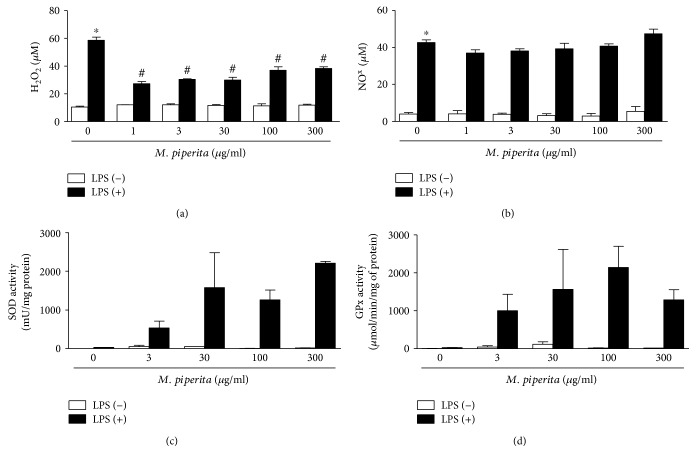
Effect of peppermint LHAE on H_2_O_2_ and NO release. H_2_O_2_ (a) and NO^x^ (b) concentrations in supernatant samples of cultured peritoneal macrophages. SOD (c) and GPx (d) activities in cultured peritoneal macrophages. Cells were pretreated with peppermint LHAE (1–300 *μ*g/ml, in 1% DMSO in PBS) and stimulated with LPS for 24 h. Vehicle-treated cells were used as controls. Data are expressed as mean ± SEM. ^∗^*p* < 0.05 compared with vehicle-treated cells; ^#^*p* < 0.05 compared with LPS-treated cells.

Figures 3(a) and 3(b) show the measured concentrations of H_2_O_2_ and NO, respectively, in supernatant samples from macrophages incubated or not with LPS and LHAE. Incubation of macrophages with LPS triggered the release of both H_2_O_2_ and NO by these cells, with fold increases of 5.6 and 10.0, respectively, for LPS-treated cells in comparison with vehicle controls. H_2_O_2_ but not NO release was reduced (34.6–53.4%) in LHAE-treated macrophages.

### 3.5. SOD and GPx Activities Are Increased in LPS-Stimulated Macrophages Treated with Peppermint LHAE

Figures 3(c) and 3(d) show the measured activities of SOD and GPx in cultured macrophages. Peppermint LHAE increased the activation of both enzymes in LPS-treated macrophages in comparison with vehicle controls. SOD activity was increased by 15.3–63.5-fold (Figure 3(c)), whilst GPx activity was raised by 34.4–73.6-fold (Figure 3(d)).

## 4. Discussion

*M. piperita* was previously suggested to have antimicrobial activity against both Gram-negative and Gram-positive bacteria [12], in addition to presenting with antioxidant potential *in vitro* [12, 17, 24]. *In vivo* anti-inflammatory actions were also reported for this plant in murine models of infection and inflammation. However, little is known on the modulatory effects of this plant in inflammatory cells. Here, we investigated the effects of a peppermint LHAE on cultured macrophages stimulated or not with LPS from *K. pneumoniae*. We found that this extract is able to modulate macrophage responses to LPS.

*Mentha* spp. effects on macrophage viability *in vitro* have been suggested to be concentration dependent. Indeed, RAW264.7 macrophage viability was previously shown not to be affected by treatment with *M. piperita* essential oil at concentrations as high as 100 *μ*g/ml [16]. On the other hand, extracts from different *Mentha* species were found to be cytotoxic in both macrophage and monocyte cell lines when assessed at concentrations >200 *μ*g/ml [25]. Thus, we initially evaluated the effects of LHAE on peritoneal macrophage viability. LPS-stimulated cells had their viability increased when incubated with LHAE in comparison with LPS controls. This was observed for the smallest concentrations tested and did not affect macrophage's ability to phagocytose when stimulated with LPS. On the other hand, at higher concentrations (≥100 *μ*g/ml), LHAE caused cytotoxicity in cells not stimulated with this endotoxin. Additional effects were also observed for LPS-untreated cells, as they presented increased phagocytosis. To the best of our knowledge, we present here the first evidence on that *M. piperita* affects the viability and phagocytosis of LPS-stimulated murine peritoneal cells. This set of results allows us to suggest that *M. piperita* effects on macrophage may be not only dependent on concentration but also on the culture conditions (presence versus absence of LPS).

In a recent study by Sun et al. [16], a peppermint essential oil reduced LPS-induced NO production by naïve RAW264.7 macrophages at similar concentrations to those tested for LHAE herein. A similar result was observed for an aqueous extract from *Mentha haplocalyx* when incubated with LPS-stimulated macrophages [26]. These studies and others [12, 17, 24] also suggested an antioxidant potential for peppermint and other plants from the same genus. We found that H_2_O_2_, but not NO production, was decreased in LHAE-treated cells stimulated with LPS at concentrations as low as 1 *μ*g/ml. The same cells presented increased SOD and GPx activities, as key antioxidant enzymes. Increased SOD activity was previously reported in mice treated with peppermint aqueous extract [27]. More recently, peppermint essential oil was shown to act as a scavenger of hydroxyl radicals and to be an antioxidant at concentrations ≥200 *μ*g/ml [16]. These results allow us to suggest that peppermint antioxidant actions on macrophages may be due to increased activation of SOD and GPx, which in turn, leads to decreased H_2_O_2_ production by these cells. These evidences, in addition to recent reports on that peppermint LHAE increases serum concentrations of anti-inflammatory cytokines in *Schistosoma mansoni*-induced infection [19], indicate an important anti-inflammatory action for *M. piperita.*

In regards to NO production, our results contradicted those described for *Mentha* spp. in the literature [28] as LHAE did not affect its levels upon macrophage stimuli with LPS. However, the inhibitory effects of *Mentha* spp. on NO release by LPS-stimulated macrophages were shown for hexane and ethyl acetate fractions [28], in addition to aqueous extract [26], suggesting that compounds found in different fractions and extractions of *Mentha* spp. may present different actions on NO production.

Different compounds were detected in essential oils obtained from peppermint leaves in previous studies [12, 17, 24, 29]. *M. piperita* antioxidant actions were previously suggested to be due to the presence of phenolic constituents in its leaves including rosmarinic acid and different flavonoids such as rutin, naringin, eriocitrin, luteolin, and hesperidin [30–33]. Here, HPLC analysis of the peppermint LHAE detected some peaks that coeluted with pure ursolic acid, epicatechin, caffeic acid, rutin, quercetin, naringenin, and kaempferol. These compounds were previously shown to act as anti-inflammatory and/or antioxidants [30, 33, 34]. It is possible that all these compounds contribute to the modulatory actions of LHAE observed in our study. However, we observed an unexpected lack of effect for LHAE on NO release by LPS-stimulated cells. This was rather surprising as it's detected compounds are known as potent inhibitors of NO production [35–40]. On the other hand, ursolic acid effects on NO release by macrophages are controversial and may be concentration dependent. Indeed, some evidences suggest this compound increases NO production by both infected [41] and resting [42] macrophages, whilst others show ursolic acid inhibits NO release by LPS-stimulated cells [43]. We suggest that, although the different compounds detected in the LHAE may contribute synergistically to its antioxidant effects, it is possible they counteract each other's abilities to stimulate or inhibit NO production by macrophages depending on their bioavailability in the extract.

**Figure 4:**
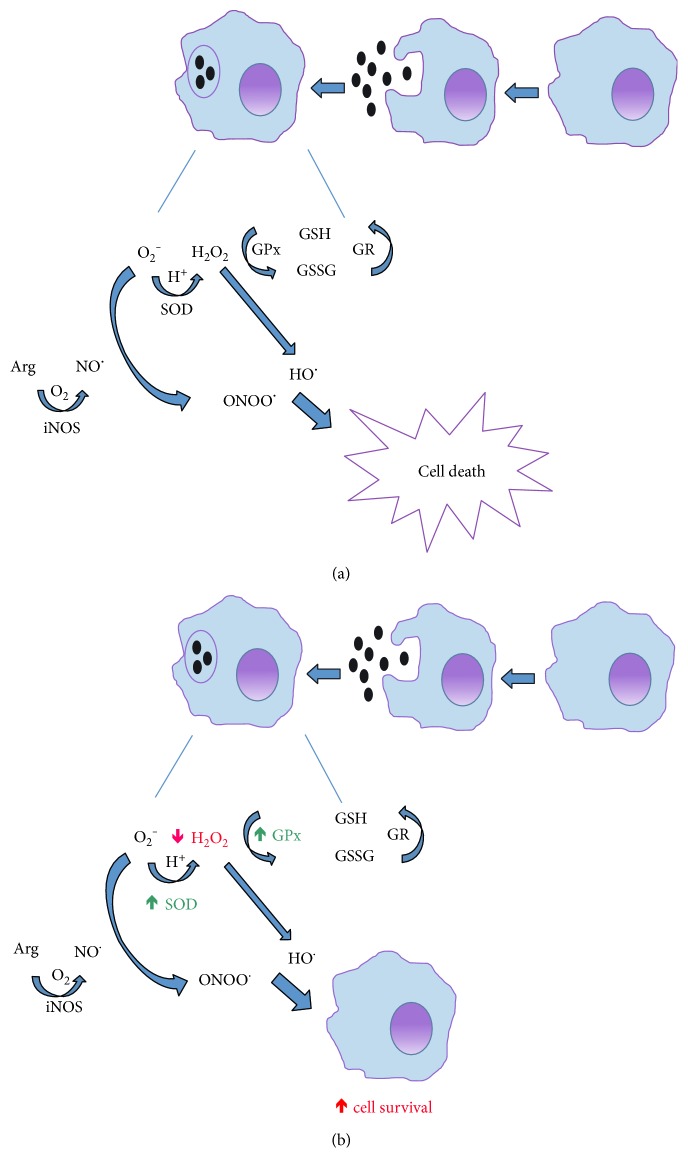
Mechanisms of action of LHAE on macrophage-mediated responses. (a) As part of the host's immune response during bacterial infections, macrophages are activated by bacteria-derived products, such as lipopolysaccharide (LPS); and as a result of this activation, reactive species are formed. Superoxide (O_2_^−^) anion produced by NADPH oxidase is converted to hydrogen peroxide (H_2_O_2_) by superoxide dismutase (SOD). H_2_O_2_ can, in turn, be further reduced to H_2_O by glutathione peroxidase (GPx) or even render hydroxyl radical (HO^.^), a much more potent oxidant that can lead to diminished cell survival via peroxidation (and further breakdown) of lipids, as well as oxidation of protein and DNA bases. In parallel, nitric oxide (NO^.^) continuously produced by inducible NO synthase (iNOS) can react with O_2_^−^ and form peroxynitrous acid (ONOOH) which, after homolytic breakdown, can also render HO^.^in addition to the highly reactive nitro (NO_2_^.^) radical (a potent modifier of proteins and lipids), thus potentiating cell death. (b) The incubation of LPS-stimulated macrophages with LHAE does not affect NO formation but rather increases SOD and GPx activities (thus lowering O_2_^−^ and H_2_O_2_ availability). As a consequence, OH and/or NO_2_^.^formation is avoided, thus improving macrophage survival.

Overall, our data show that the peppermint LHAE modulates macrophage-mediated inflammatory responses, by stimulating the antioxidant pathway in these cells (Figure 4). These effects may be beneficial when the excessive activation of macrophages contributes to tissue damage in diseases in which there is an unbalanced oxidative stress, such as those of infectious nature.

## Figures and Tables

**Table 1:** Registered retention times obtained by HPLC analysis of the peppermint LHAE. Retention times in minutes were registered for each peak.

Peak number	Retention time in min	Compound
1	13.8	Ursolic acid
2	14.5	Epicatechin
3	16.3	Caffeic acid
4	23.6	Rutin
5	27.6	Quercetin
6	33.5	Naringenin
7	37.8	Kaempferol

## References

[1] Gleeson L. E., Sheedy F. J. (2016). Metabolic reprogramming & inflammation: fuelling the host response to pathogens. *Seminars in Immunology*.

[2] Keyel P. A., Heid M. E., Salter R. D. (2011). Macrophage responses to bacterial toxins: a balance between activation and suppression. *Immunologic Research*.

[3] Salomao R., Brunialti M. K., Rapozo M. M., Baggio-Zappia G. L., Galanos C., Freudenberg M. (2012). Bacterial sensing, cell signaling, and modulation of the immune response during sepsis. *Shock*.

[4] Cerceo E., Deitelzweig S. B., Sherman B. M., Amin A. N. (2016). Multidrug-resistant Gram-negative bacterial infections in the hospital setting: overview, implications for clinical practice, and emerging treatment options. *Microbial Drug Resistance*.

[5] Kaye K. S., Pogue J. M. (2015). Infections caused by resistant gram-negative bacteria: epidemiology and management. *Pharmacotherapy*.

[6] Roger T., Delaloye J., Chanson A.-L., Giddey M., Le Roy D., Calandra T. (2012). Macrophage migration inhibitory factor deficiency is associated with impaired killing of gram-negative bacteria by macrophages and increased susceptibility to *Klebsiella pneumoniae* sepsis. *Journal of Infectious Diseases*.

[7] Flint A., Stintzi A., Saraiva L. M. (2016). Oxidative and nitrosative stress defences of Helicobacter and Campylobacter species that counteract mammalian immunity. *FEMS Microbiology Reviews*.

[8] Grant S. S., Hung D. T. (2013). Persistent bacterial infections, antibiotic tolerance, and the oxidative stress response. *Virulence*.

[9] Kaufmann S. H., Dorhoi A. (2016). Molecular determinants in phagocyte-bacteria interactions. *Immunity*.

[10] Nathan C., Cunningham-Bussel A. (2013). Beyond oxidative stress: an immunologist’s guide to reactive oxygen species. *Nature Reviews: Immunology*.

[11] Lawrence B. M. (2006). *Mint: The Genus Mentha*.

[12] da Silva Ramos R., Rodrigues A. B., Farias A. L. (2017). Chemical composition and in vitro antioxidant, cytotoxic, antimicrobial, and larvicidal activities of the essential oil of *Mentha piperita* L. (Lamiaceae). *The Scientific World Journal*.

[13] Golestani M. R., Rad M., Bassami M., Afkhami-Goli A. (2015). Analysis and evaluation of antibacterial effects of new herbal formulas, AP-001 and AP-002, against *Escherichia coli* O157: H7. *Life Sciences*.

[14] Husain F. M., Ahmad I., Khan M. S. (2015). Sub-MICs of *Mentha piperita* essential oil and menthol inhibits AHL mediated quorum sensing and biofilm of Gram-negative bacteria. *Frontiers in Microbiology*.

[15] Jain D., Pathak N., Khan S. (2011). Evaluation of cytotoxicity and anticarcinogenic potential of *Mentha* leaf extracts. *International Journal of Toxicology*.

[16] Sun Z., Wang H., Wang J., Zhou L., Yang P. (2014). Chemical composition and anti-inflammatory, cytotoxic and antioxidant activities of essential oil from leaves of *Mentha piperita* grown in China. *PLoS One*.

[17] Yadegarinia D., Gachkar L., Rezaei M. B., Taghizadeh M., Astaneh S. A., Rasooli I. (2006). Biochemical activities of Iranian *Mentha piperita* L. and *Myrtus communis* L. essential oils. *Phytochemistry*.

[18] Bouari C., Bolfa P., Borza G., Nadăş G., Cătoi C., Fiţ N. (2014). Antimicrobial activity of *Mentha piperita* and *Saturenja hortensis* in a murine model of cutaneous protothecosis. *Journal of Medical Mycology*.

[19] Dejani N. N., Souza L. C., Oliveira S. R. (2014). Immunological and parasitological parameters in *Schistosoma mansoni*-infected mice treated with crude extract from the leaves of *Mentha* x *piperita* L. *Immunobiology*.

[20] Fernandes E. S., Liang L., Smillie S. J. (2012). TRPV1 deletion enhances local inflammation and accelerates the onset of systemic inflammatory response syndrome. *Journal of Immunology*.

[21] Mendes S. J., Sousa F. I., Pereira D. M. (2016). Cinnamaldehyde modulates LPS-induced systemic inflammatory response syndrome through TRPA1-dependent and independent mechanisms. *International Immunopharmacology*.

[22] Ukeda H., Maeda S., Ishii T., Sawamura M. (1997). Spectrophotometric assay for superoxide dismutase based on tetrazolium salt 3′-{1-[(phenylamino)-carbonyl]-3, 4-tetrazolium}-bis (4-methoxy-6-nitro) benzenesulfonic acid hydrate reduction by xanthine–xanthine oxidase. *Analytical Biochemistry*.

[23] Paglia D. E., Valentine W. N. (1967). Studies on the quantitative and qualitative characterization of erythrocyte glutathione peroxidase. *Journal of Laboratory and Clinical Medicine*.

[24] Uribe E., Marín D., Vega-Gálvez A., Quispe-Fuentes I., Rodríguez A. (2016). Assessment of vacuum-dried peppermint (*Mentha piperita* L.) as a source of natural antioxidants. *Food Chemistry*.

[25] Brahmi F., Hadj-Ahmed S., Zarrouk A. (2017). Evidence of biological activity of *Mentha* species extracts on apoptotic and autophagic targets on murine RAW264. 7 and human U937 monocytic cells. *Pharmaceutical Biology*.

[26] Liao H., Banbury L. K., Leach D. N. (2007). Elucidation of danzhixiaoyao wan and its constituent herbs on antioxidant activity and inhibition of nitric oxide production. *Evidence-based Complementary and Alternative Medicine*.

[27] Samarth R., Panwar M., Kumar A. (2006). Modulatory effects of *Mentha piperita* on lung tumor incidence, genotoxicity, and oxidative stress in benzo[*a*]pyrene-treated Swiss albino mice. *Environmental and Molecular Mutagenesis*.

[28] Karimian P., Kavoosi G., Amirghofran Z. (2013). Anti-inflammatory effect of *Mentha longifolia* in lipopolysaccharide-stimulated macrophages: reduction of nitric oxide production through inhibition of inducible nitric oxide synthase. *Journal of Immunotoxicology*.

[29] Mogosan C., Vostinaru O., Oprean R. (2017). A comparative analysis of the chemical composition, anti-inflammatory, and antinociceptive effects of the essential oils from three species of *Mentha* cultivated in Romania. *Molecules*.

[30] Dorman H., Koşar M., Başer K., Hiltunen R. (2009). Phenolic profile and antioxidant evaluation of *Mentha* x *piperita* L. (peppermint) extracts. *Natural Product Communications*.

[31] McKay D. L., Blumberg J. B. (2006). A review of the bioactivity and potential health benefits of peppermint tea (*Mentha piperita* L.). *Phytotherapy Research*.

[32] Pérez M. G. F., Rocha-Guzmán N. E., Mercado-Silva E., Loarca-Piña G., Reynoso-Camacho R. (2014). Effect of chemical elicitors on peppermint (*Mentha piperita*) plants and their impact on the metabolite profile and antioxidant capacity of resulting infusions. *Food Chemistry*.

[33] Sroka Z., Fecka I., Cisowski W. (2005). Antiradical and anti-H_2_O_2_ properties of polyphenolic compounds from an aqueous peppermint extract. *Zeitschrift für Naturforschung Teil C: Biochemie, Biophysik, Biologie, Virologie*.

[34] Guruvayoorappan C., Kuttan G. (2007). Rutin inhibits nitric oxide and tumor necrosis factor-alpha production in lipopolysaccharide and concanavalin-a stimulated macrophages. *Drug Metabolism and Drug Interactions*.

[35] Hamalainen M., Nieminen R., Vuorela P., Heinonen M., Moilanen E. (2007). Anti-inflammatory effects of flavonoids: genistein, kaempferol, quercetin, and daidzein inhibit STAT-1 and NF-κB activations, whereas flavone, isorhamnetin, naringenin, and pelargonidin inhibit only NF-κB activation along with their inhibitory effect on iNOS expression and NO production in activated macrophages. *Mediators of Inflammation*.

[36] Kumar P., Abraham A. (2017). Inhibition of LPS induced pro-inflammatory responses in RAW264. 7 macrophage cells by PVP coated naringenin nanoparticle via down regulation of NF-κB/P38MAPK mediated stress signaling. *Pharmacological Reports*.

[37] Lyu S.-Y., Park W.-B. (2005). Production of cytokine and NO by RAW 264.7 macrophages and PBMC in vitro incubation with flavonoids. *Archives of Pharmacal Research*.

[38] Mu M. M., Chakravortty D., Sugiyama T. (2001). The inhibitory action of quercetin on lipopolysaccharide-induced nitric oxide production in RAW 264.7 macrophage cells. *Journal of Endotoxin Research*.

[39] Song Y. S., Park E. H., Hur G. M. (2002). Caffeic acid phenethyl ester inhibits nitric oxide synthase gene expression and enzyme activity. *Cancer Letters*.

[40] Zhang J., Xu L. X., Xu X. S., Li B. W., Wang R., Fu J. J. (2014). Synthesis and effects of new caffeic acid derivatives on nitric oxide production in lipopolysaccharide-induced RAW 264.7 macrophages. *International Journal of Clinical and Experimental Medicine*.

[41] Lopez-Garcia S., Castaneda-Sanchez J. I., Jimenez-Arellanes A. (2015). Macrophage activation by ursolic and oleanolic acids during mycobacterial infection. *Molecules*.

[42] You H. J., Choi C. Y., Kim J. Y., Park S. J., Hahm K. S., Jeong H. G. (2001). Ursolic acid enhances nitric oxide and tumor necrosis factor-α production via nuclear factor-κB activation in the resting macrophages. *FEBS Letters*.

[43] Kim M. H., Kim J. N., Han S. N., Kim H. K. (2015). Ursolic acid isolated from guava leaves inhibits inflammatory mediators and reactive oxygen species in LPS-stimulated macrophages. *Immunopharmacology and Immunotoxicology*.

